# Letter from Dr. Beaugrand—No. II

**Published:** 1839-08-17

**Authors:** 


### FOREIGN CORRESPONDENCE.

### LETTER FROM DR. BEAUGRAND—No. II.

*The late Concours for the Chair of Materia Medica and Therapeutics in the Faculty of Medicine, of Paris—Dr. Requin's Essay on Purgatives.*

PARIS, *June 18th*, 1839.

To the Editors of the Medical Examiner.

It has been with justice, and not lately for the first time, remarked, that subjects imposed as tasks, at a *concours*, for example, are generally but indifferently treated, in a style unworthy the reputation of the authors, and inferior to the productions which they have published under the sole inspiration of their genius. This is the secret of the feebleness of the papers which competed for the prize of the Royal Academy of Surgery—a feebleness which strikingly contrasts with the ability of the immortal memoirs of the same society. These reflections apply to the merits of the theses of the *concours* now opened before the faculty of medicine of Paris, for the chair of therapeutics and materia medica. It will be readily understood that these theses, subjected to the discussion of the competitors, do not escape a very rigorous criticism, which scrutinizes every error and every omission. It must be borne in mind, that but twelve days are allowed for the composition and printing of these essays. Is it surprising, then, if they bear evidence of precipitation, of the anxiety of every description which assails the unfortunate competitors, in this important and decisive crisis? And yet, such is the merit of some of these essays, as well as the importance of the subject discussed, that they are well worth an attentive examination.

The first thesis to which I wish to direct the notice of your readers, is that of Dr. Requin, a physician favourably known, from many useful productions, and a depth of erudition and fluency of elocution, displayed in repeated *concours*. Dr. Requin had assigned to him one of the most interesting subjects in therapeutics, which was announced by the judges with the following caption—*Purgatives and their principal uses.*

This subject, besides its intrinsic importance, has the particular interest of being completely *à l'ordre du jour*. During the reign of the doctrines styled physiological, purgatives were nearly banished from our French materia medica; and the epithets, incendiary and assassin, were deemed scarcely severe enough to characterize the audacious practitioner, who ventured to cure his patients by such remedies. Now, that this enthusiasm has evaporated, and that the theory of irritation has been reduced to proper limits, the stimulating remedies, including purgatives, have been restored to favour, and recovered their old and familiar, but lately suspended rights. During the reign of proscription, however, the sciences of chemistry and pharmacy had been making continual advances, and purgatives were, in their turn, undergoing the reform which has regenerated our whole modern materia medica. From the shops of the apothecary have been banished those complicated preparations, of which our ancestors were so fond. We have obtained the active principles of a number of vegetable substances,—in a word, there has been a simplification of remedies, as regards both their composition and their mode of administration. There was, besides these, another improvement to be accomplished, which was, properly to appreciate the physiological action of purgative substances, and to lay down with precision the indications for their employment, and the circumstances under which they are to be regarded as counter-indicated. These were the points, in relation to purgatives, which Dr. Requin undertook to elucidate; and we cannot hesitate to say, that he has accomplished his task with equal method, exactness, and talent. His thesis constitutes a pamphlet of eighty pages, divided into five chapters. In the first, he gives us a general idea of purgatives; in the second, he classifies and arranges them according to their degree of activity; the third he devotes to the study of their physiological action; the fourth to an examination of the modes of preparing them; and in the fifth, by far the most important, he lays down the indications for their employment. We propose to examine the questions successively under these different points of view, without following very closely the divisions selected by the author.

What is a purgative? The etymology of the word is readily traced to the old humoral pathology; for, to purge, means, in other words, to evacuate the impure humours which may be collected in the economy. At the present day, authors are agreed in classifying, under the head of purgatives, all remedies, the evident result of which is, to excite alvine evacuations, more or less abundant. This definition is that which Dr. Requin has adopted; he thus gets rid of the whole tribe of emetics, the effect of which is to produce vomiting. It is needless here to dwell upon the unequal parts which the three kingdoms of nature play in furnishing the materia medica with purgatives; it is well known that to vegetables and minerals, is almost exclusively confined the property of purgation. As for the mode of preparation of these substances, it is equally well known how infinitely they may be varied in doses and proportions, without approaching the vast polypharmacy of the Galenic school.

Purgatives are not all endowed with the same energy; in fact, some, administered even in large doses, increase but in a feeble degree the peristaltic motion of the intestines,—while others, in moderate or very small doses, produce abundant excretions, accompanied by phenomena, in various degrees, of local and general irritation; others, again, act with a violence which may produce actual inflammation. Hence we have the triple division of purgatives into *laxatives*, *cathartics*, and *drastics*. It must not, however, be forgotten, that circumstances may influence the action of remedies, and that in a particular person a laxative will operate with extreme violence, while, in another, a drastic will determine a mild and moderate action.

We consider as true *laxatives*, substances of an organic nature, which are, to a certain point, digestible and alimentary, but which possess likewise a purgative influence. This influence is consecutive upon an actual indigestion, which causes the intestinal contents to be thrown out altogether, or in part. It is only, then, from their action as foreign bodies, that laxatives appear to excite the irritability of the intestinal canal, thereby slightly accelerating the peristaltic motion, usually, however, without any notable increase of the secretory irritation.

*Cathartics* have the character neither of laxatives nor of drastics. In the first place, on the one hand, cathartics are, in no degree, susceptible of digestion; in the second place, on the other, although the greater number of them carry their action beyond the simple expulsion of fecal matter, producing intestinal irritation of a degree to determine severe colics and an abundant discharge of secretory matter, there are none of them possessing essentially the property of true drastics—of inflaming any tissue with which they come in contact.

*Drastics* are distinguished by a property essentially inflammatory: their contact, sufficiently prolonged, inflames the tissues to which they are applied. As soon, then, as they are introduced into the stomach, they cause the most lively irritation. In small doses, they produce violent purgation, with sharp pains, nausea, and sometimes vomiting. If given in quantities imprudently large, they act as poisons, and threaten to extinguish life. This is one of their peculiar characteristics.

A very important point to determine, is the physiological action of purgatives; for upon this depends their therapeutical application. With what propriety can a remedy be exhibited to a sick man, if we are ignorant of the precise effect it would produce upon him in health?

Purgation is neither a mechanical nor chemical, but it is a vital phenomenon—a special modification of the intestinal functions. Certain remedies act by a simple local irritation, and, in some measure, as foreign bodies, and these include the great body of purgatives; while others possess virtues which are really specific, and act upon the intestines, whatever be the mode of their introduction into the system. We have a striking example of this in the action of croton oil, or of aloes, employed by means of friction, after the endermic method.

With the local effects of purgatives every one is familiar: to stools, simply stercoraceous, succeed those which are liquid, varying in number, and in character, according to the individual and to the substances employed. The intestinal irritation, which determines a more abundant secretion of the normal humours, and an increase of activity in the peristaltic motions, has an intensity and duration much less than was maintained by Broussais, in furtherance of his own doctrines. Nothing but the most obstinate blindness—I may say fanaticism, on the part of his followers—could fail to recognise this important truth. It is well established that the purgative irritation may be repeated tolerably often, and at short intervals, for a pretty long period, without any of the bad results, which it was the fashion some years since to believe. These are facts of the highest practical importance, which the theorists must admit, explain the operation as they may. It must be borne in mind, nevertheless, that drastics are not to be incautiously employed. Due limits are to be observed, and one extreme is not to be exchanged for another.

We shall not stop to examine whether every portion of the digestive system has its special stimulant. It seems to us that the assertions which have been made upon this point, are somewhat hypothetical, and need verification. It is, however, a well established fact, that aloes has a specific action upon the rectum, which may be turned to account in suppressed hæmorrhoids, and other affections.

Besides local effects, purgatives produce likewise phenomena of a general character, which consist chiefly in rapid debility, and a tendency to syncope. The same results happen in diarrhœas and colics arising spontaneously. But, what is not a little singular, is, that, after these first phenomena, a marked change occurs in the pulse, which loses strength and hardness. We have therefore a *moderation of the febrile condition.* Finally, purgation diminishes the other secretions, and, as a consequence of the depletion which it produces, it promotes absorption and acts in the same manner as bleeding; that is, it facilitates the resolution of inflammatory engorgements.

Improperly employed, evacuating remedies, may produce serious consequences. In the first place, they may bring about constipation, inasmuch as they in some manner blunt the natural excitability of the intestine, for which the normal stimulation of the presence of fecal matter ought to be sufficient. In the second place, the *constant* repetition of purgative evacuations may finally radically impair the function of nutrition, and prostrate the entire economy.

As for the mode of administration of purgatives, and the precautions to be taken before, and during their action, these are trifles, to be found in any work on the subject, and of familiar and domestic knowledge. We shall merely mention here, a point upon which the author of the thesis properly dwells—that the infant at the breast may readily be purged by administering rhubarb or jalap to the nurse. But it is a subject little understood, and little studied, although important to ascertain, which among the purgatives soonest transmit their action, or rather themselves, by this mode of exhibition.

I now come to the fifth and last chapter of the division, and the most interesting of the thesis, in a practical point of view, that which treats of the indications proper for the employment of purgatives. The author has not followed out all the developments of which the subject allowed, but he has, nevertheless, presented a very good summary of the actual state of our knowledge in this particular. The chapter is divided into two parts; the first treats of the *rational*, the second of the *empirical* indications. The rational indications are all those which are founded upon the consideration of the physiological effects of purgative remedies. They are the results of a process of reasoning, based, on the one hand, upon the physiological, and on the other, on the therapeutic effects of those substances.

1st. Considering purgatives, in the first place, merely as evacuants of the alimentary canal, we find them, in this point of view, proper to fulfil various indications. They may render essential service, from the simple fact of their expelling from the economy, fecal matter, which is acting as a true foreign body, and which the intestine itself is often inadequate to get rid of. This is the *eccoprotic* indication. In France, this indication is fulfilled, during the course of acute affections, by administering to the patient simple injections. In England, they act more boldly, and give slight purgatives by the mouth; nor do their practitioners find any reason to regret such a course. The undue accumulation of fecal matter in the intestines, often simulates the symptoms of peritonitis or of enteritis; but the tumefaction and hardness of the cœcal region, and the absence of stools, leave no difficulty in recognising the true nature of the affection, which yields, magically, almost, to a single purgative dose. Dr. Requin has repeatedly seen much time lost—he frankly admits that he has lost time himself—by treating with baths, leeches, emollient poultices, &c., patients who require simple purging. As eccoprotics, the weakest purgatives must be employed; the indication, however, once recognised, if the weaker purgatives fail to act, the more energetic must unhesitatingly be employed, croton oil, if necessary. In chronic affections, on the contrary, purging must be resorted to with caution, as it would but increase the intestinal sluggishness. Under these circumstances, the constipation is to be combatted as much as possible by hygienic remedies.

2d. The value of purgatives to meet the *vermifuge* indication is well established. In this case those purgatives are to be preferred which seem to exercise a specially noxious influence upon the intestinal worms. It is even proper to postpone the purgative, until after the exhibition of some substance which destroys the parasitic animal.

3d. The *anti-toxical* indication which is fulfilled by causing the evacuation per anum of poisonous matter contained in the intestine, will be disputed by no one. There are, besides, circumstances, in which the advantageous action of purgatives has been explained, by their causing the expulsion of noxious matter, secreted by the intestines themselves. It is thus that it has been attempted to explain the success of the treatment in certain epidemics of puerperal peritonitis, of dysentery, and in typhoid fever. These, however, are mere hypotheses, for it might with equal propriety be said that the irritative action of the purgatives, changes the character of the irritation with which the intestine is morbidly affected, and restores it to a normal condition. Neither of these speculations is better established than the other. What is very certain, is, that the purgative treatment is successful in the cases mentioned.

4th. But purgatives are not merely local evacuants of the intestinal canal; they act also, as *general evacuants* upon the whole economy. The immediate abatement of the symptoms, which follows their administration, accounts readily for their success in the management of severe fevers. On the other hand, the increase of the intestinal secretion, from the remedies in question, bringing about a diminution of other secretions, it will be understood, how usefully they may be employed; for example, in drying up the milk secretion of recently delivered women, in arresting any abnormal flux whatever, in the management of old ulcers or other discharges. Repeated alvine evacuations favor absorption, as we have already observed; the advantages to be derived from them in dropsies are obvious, enormous serous effusions being rapidly made to disappear, under the establishment of a copious diarrhœa.

5th. Purgatives may further be considered as *irritants* of the intestinal canal. In this capacity, they act on the well known principle of revulsion. They are, therefore, to be unhesitatingly resorted to in cerebral and pectoral irritations, unless counter-indicated by some idiosyncrasy; but, as we have already said more than once, and again repeat, the time has gone by for absurd terrors about gastro-intestinal irritation. We have already noticed the specific action of aloes upon the inferior extremity of the large intestine; hence their use in restoring suppressed hæmorrhoids, and indirectly stimulating the uterus, in dysmenorrhœa.

After the *rational* indications, (in which the effects of purgatives are explained by their physiological action,) come the *empirical* indications—that is, those which have no other foundation than success established by practice, which, after all, is as good as any other. The purgative treatment is successful in painter's colic, in gastro-intestinal derangements, in certain dysenteries, and in typhoid fever. The explanation of their modus operandi has been attempted by various hypotheses, but, whatever be the explanation, the fact is indisputable.

The treatment by emetics and purgatives, constitutes the old method, styled *de la Charité*, and its success was prompt and general. It is well known, that, in the few fatal cases from this treatment, the autopsies revealed no sort of inflammation, or even irritation of the intestines—an unanswerable refutation of the objections to purgatives.

We now come to the subject of typhoid fever, a subject, "adhuc sub judice." Formerly employed in the putrid and malignant affections, which we have converted into our modern typhoid fever, purgatives have of late been theoretically regarded as incendiary and mortal, by the ultra-solidists of the school of Broussais, who could see in this affection only a gastro-enteric inflammation. But, within a few years, we have taken up better ideas, and looked to other modes of treatment, besides depletion, in combatting this affection. In 1832, M. Delarroque began to employ an emetico-cathartic treatment, principally cathartic, in typhoid fever; and appealing to the statistics of a very large practice, he proclaimed the purgative to be the most successful treatment, and that it should be indiscriminately prescribed to the exclusion of depletion, in this disease. At the same time, M. Delarroque attempted to revive the old doctrine of a noxious putrid principle in the economy, which it was his object to expel. But, with hypotheses we have little concern. After the example of M. Delarroque, several physicians connected with hospitals, Drs.

Piédagnel, Pras, Louis, Andral, &c., tried the evacuant plan of treatment. In a report, on the subject, made by Professor Andral, in 1837, to the Royal Academy of Medicine, are given the principal results obtained by the professor and several other physicians.

Dr. Andral submitted to the treatment of M. Delarroque forty-eight patients, in whom the diagnosis of typhoid fever was clearly established, and lost eight of them, being a mortality of one in six—a result less favourable than that of M. Delarroque, who lost only a tenth of his patients. These results, however, should be regarded as very satisfactory, compared with those observed by Dr. Andral, after a treatment of moderate bleedings. Of twenty-seven patients, six died,—that is, one to four and a half,—and of those who were treated by bleeding and purging combined, there was a mortality of one-third. The experience of Dr. Louis, on the contrary, gave but a very trifling advantage to the purgative treatment. He lost no less than a tenth of his patients, (three out of thirty-one, or one to ten and a third,) whilst with the rational or symptomatic treatment, was one to nine and five-elevenths, (eleven in one hundred and four.) But is the inference from these data, that the treatment in question is to be employed indiscriminately, to the exclusion of every other? Surely not; there are cases in which it would be hurtful. Here, as in all diseases, symptoms and idiosyncracies must be regarded.

To terminate these remarks upon the employment of the evacuant method in severe fevers, I shall extract from the excellent dissertation of M. Beau, an account of M. Delarroque's mode of using the remedy:—"He allows the patient to drink barley water, or lemonade, at his option; and he begins his active treatment by the exhibition of a grain or two of tartar emetic, in barley water or milk, without reference to the state of the tongue, thirst, pain, &c., in a word, without regard to the type or grade of the fever. The next day, he gives a Seidlitz powder, and repeats it daily, unless the patient becomes disgusted with it. In that case, it is replaced by cream of tartar, castor oil, in the dose of an ounce, or, still better, calomel in two consecutive doses of eight grains each. The last remedy is here exceedingly valuable, from its insipidity; but it would be imprudent to repeat it too often, for fear of salivation,—on this account, it is better not to give it, unless the patient refuse other laxatives. The different symptoms should not be the object of general attention, much less should they be treated by general or local bleeding, which should never be allowed under any circumstances, or for any object. A congestion of the lungs alone demands special attention; resolution is to be excited and promoted by means of Kermes mineral, given in a lozenge, in the dose of five or eight grains. Should it purge sufficiently, the employment of other laxatives may be suspended."

At the beginning of the letter, we remarked, that the work of Dr. Requin was one of great interest. It will tend to encourage physicians to the employment of a powerful remedy, long fallen into disuse and contempt. Such an end, obtained by an academic contest, is worth the more showy triumph arising from the production of a specious theory, or from a brilliant discussion upon speculations.

[Much of the contents of this letter may appear to be familiar truisms. But, it should be borne in mind, that, while American and British practitioners have always held fast to purging, as the sheet-anchor of their practice, it was, for a time, literally proscribed in France. Even now, after the downfall of Broussaism, though returning into use, it is the object of much professional suspicion and popular aversion. Coming from such a quarter, our correspondent's sound and able exposition of the state of our knowledge on the subject, is not without interest.—Eds.]

